# Digital biomechanical assessment of gait in patients with peripheral neuropathies

**DOI:** 10.1186/s12984-025-01694-w

**Published:** 2025-07-13

**Authors:** Clara Tejada-Illa, Jordi Pegueroles, Mireia Claramunt-Molet, Ariadna Pi-Cervera, Ainhoa Heras-Delgado, Jesus Gascón-Fontal, Sebastian Idelsohn-Zielonka, Mari Rico, Nuria Vidal, Lorena Martín-Aguilar, Marta Caballero-Ávila, Cinta Lleixà, Roger Collet-Vidiella, Laura Llansó, Álvaro Carbayo, Ana Vesperinas, Luis Querol, Elba Pascual-Goñi

**Affiliations:** 1 Institut de Recerca Sant Pau (IR SANT PAU), Barcelona, Spain; 2 Neuromuscular Diseases Unit, Department of Neurology, Hospital de la Santa Creu i Sant Pau, Universitat Autònoma de Barcelona, Sant Quintí 77 (2nd Floor), 08041 Barcelona, Spain; 3 Centro Para la Investigación Biomédica en Red en Enfermedades Raras (CIBERER), Madrid, Spain; 4 Ephion Health, Barcelona, Spain; 5 Unit of Digital Health, Eurecat, Centre Tecnològic de Catalunya, Barcelona, Spain

**Keywords:** Neuropathies, Gait disfunction, Ataxia, Steppage, Wearable devices, Biomechanical features

## Abstract

**Background:**

The clinical status and treatment response of patients with peripheral neuropathies (PNs) rely on subjective and inaccurate clinical scales. Wearable sensors have been evaluated successfully in other neurological conditions to study gait and balance. Our aim was to explore the ability of biomechanical analysis using wearable technology to monitor disease activity in PN.

**Methods:**

We conducted a single-center, longitudinal study to analyze gait parameters in PN patients and healthy controls using wearable biomechanical sensors. We used a novel technology that registers and integrates data from multiple wearable inertial sensors placed at different locations and plantar insoles. This system allows measuring kinematics, spatio-temporal parameters and plantar pressure. Patients wore the wearable system while performing the 2-min walking test (2MWT).

**Results:**

We included 37 chronic inflammatory demyelinating polyneuropathy (CIDP) patients, 3 chronic ataxic neuropathy, ophthalmoplegia, immunoglobulin M [IgM] paraprotein (CANOMAD) patients, 21 monoclonal gammopathy patients of undetermined significance associated with IgM (IgM-MGUS) patients, 7 patients with autoimmune nodopathies, 11 patients with hereditary neuropathies, and 50 healthy controls. First, we analyzed the sensor's ability to detect changes in ataxia and steppage gait severity and found significant differences in spatiotemporal and angular variables of the gait cycle. Second, we found correlations between biomechanical features and clinical scales and with the specific gait phenotype they associated with. Finally, we demonstrated that this technology is able to capture clinically significant changes in gait features over time.

**Conclusions:**

Our study provides proof-of-concept that wearable technology effectively detects and grades gait impairment, captures clinically relevant changes, and could enhance gait assessment in routine care and clinical research for patients with PN.

**Supplementary Information:**

The online version contains supplementary material available at 10.1186/s12984-025-01694-w.

## Introduction

Peripheral neuropathies (PNs) are a heterogeneous group of diseases of the peripheral nervous system [1–3] that present with diverse symptoms, including weakness, sensory disturbances, ataxia, fatigue, and pain, that frequently cause gait dysfunction [4–7]. These diseases lack objective prognostic and disease activity biomarkers [8–10]; assessments of clinical status and disease activity are based on clinical scales, which are often imprecise [11, 12], with significant ceiling and floor effects and fluctuations in stable patients [13], which are associated with substantial placebo effects [14], and fail to capture minor disease changes that could reflect ongoing nerve damage. In the long term, this imprecision results in inefficient care and, eventually, irreversible disability [15]. Thus, there is an unmet need in the field to develop less subjective outcome measures to quantify and monitor disease parameters [9, 16, 17].

In recent years, new technologies, such as portable inertial sensors that are able to measure motor capacity [18, 19] and gait, have been developed [20, 21]. These sensors are placed on the patient's body and are incorporated into insoles, bracelets, or clothing [22, 23], which allows the unbiased measurement of diverse biomechanical parameters while performing motor tasks [24, 25]. Novel wearable sensors have been successfully used to monitor gait and balance in patients with other neurological diseases, such as multiple sclerosis [26–28] and Parkinson disease [18, 19, 29, 30] and may provide greater objectivity [30] and measurability [31] in the assessment of the clinical status of neurological patients, helping overcome the limitations of current monitoring strategies [32, 33]. Moreover, the development of affordable technologies capable of automatically recognizing and monitoring disease status may improve disease care and reduce the burden on the healthcare system [34–36].

Wearable technologies have been previously evaluated in patients with PN, including diabetic peripheral neuropathy (DPN) [37, 38], inflammatory neuropathies [33], and hereditary neuropathies [31]. For instance, Brognara et al. [39] assessed postural stability in patients with DPN using a single lumbar-mounted IMU (mSway) during eight 30-s trials under varying sensory conditions. Sway parameters were analyzed alongside clinical neuropathy tests to identify markers of sensory impairment and fall risk. However, to date, no single study has investigated the utility of multiparametric wearable sensors to assess gait patterns across a heterogenous cohort of PN patients.

Our main objective was to test the ability of a set of digital biomechanical features (DBF), consisting of multiple spatiotemporal features, biomechanical angles, and plantar pressure parameters, to monitor the clinical status of patients with PNs. To achieve this goal, three objectives were developed. First, we investigated whether DBF could detect differences from healthy controls in two frequent gait phenotypes that appear in patients with PNs: gait ataxia and steppage gait. Second, we analyzed whether DBF was correlated with impairment and disability clinical scales. Third, we tested the ability of DBF to capture clinically significant changes over time.

## Methods

### Patients

Patients with PNs followed in the Neuromuscular Diseases Unit of our center were included in the study. The mean age was 62.2 years, and 51 patients (64.6%) were male. We recruited 37 chronic inflammatory demyelinating polyneuropathy (CIDP) patients (46.8%), 3 chronic ataxic neuropathy, ophthalmoplegia, immunoglobulin M [IgM] paraprotein (CANOMAD) patients (3.8%), 21 monoclonal gammopathy patients of undetermined significance associated with IgM (IgM-MGUS) patients (26.5%), 7 patients with autoimmune nodopathies (8.9%), 11 patients with hereditary neuropathies (13.9%), and 50 healthy controls. Patients were classified by the evaluating neurologist according to their gait pattern into three gait groups (ataxia gait, steppage or normal gait). Additionally, the severity of ataxia and steppage (mild, moderate, or severe) was classified according to prespecified criteria (Supplementary Table 1).

This study was conducted according to a protocol approved by the Ethics Committee of the Hospital de la Santa Creu i Sant Pau (code IIBSP-NMI-2019-107) in accordance with the declaration of Helsinki. All patients provided written informed consent to participate in the study.

### Protocol of the study

**Fig. 1:**
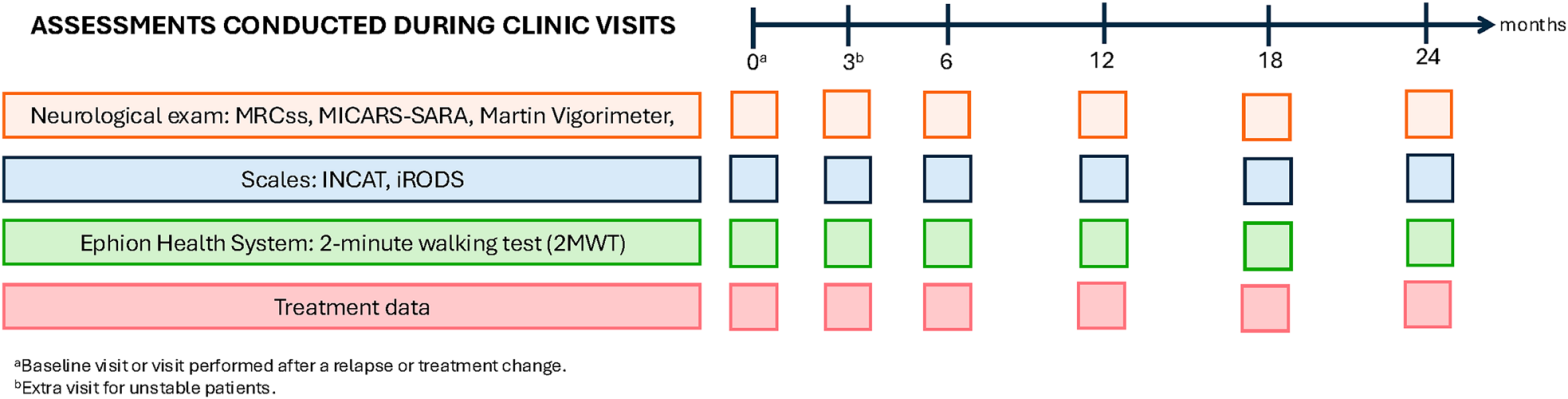
Protocol of the study. Monitoring visits were performed every 6 months for 2 years for those patients who remained stable and for those who presented a relapse or a treatment change, a 3-month visit (3*) was performed after that date. MRCss: Medical Research Council sum score; INCAT: Inflammatory Neuropathy Cause and Treatment; iRODS: Inflammatory Rasch-built Overall Disability Scale; MICARS-SARA: Modified Internacional Cooperative Ataxia Rating Scale and Scale for the Assessment and Rating of Ataxia

Patients were monitored for two years. Visits were scheduled every 6 months for stable patients. Patients who experienced a relapse or a treatment change, a visit was conducted at the time of the event followed by an additional visit 3 months after the event. Once clinical stability was re-established, the regular 6-month monitorization was resumed. The protocol scheme followed through the visits is summarized in Fig. 1. During the visits, the evaluating neurologists performed a thorough neurological evaluation including the following scales or scores: Medical Research Council sum score (MRCss), Inflammatory Neuropathy Cause and Treatment (INCAT), Modified Internacional Cooperative Ataxia Rating Scale and Scale for the Assessment and Rating of Ataxia (MICARS-SARA) and grip strength testing via a Martin vigorimeter. After the neurological evaluation, patients completed the Inflammatory Rasch-built Overall Disability Scale (iRODS) questionnaire. Finally, a single 2-min walking test (2MWT) was performed while the participants wore all the biomechanical sensors.

### Wearable technology

We used the Ephion Mobility system [40] (Ephion Health, Barcelona, Spain), which integrates multiple parameter sensors, including 7 inertial sensors (Movesense, Vantaa, Finland [41]) placed at different locations (ankles, thigh, and chest) and insoles with inertial and plantar pressure sensors (Moticon, Munich, Germany [42]). The full protocol included pants with integrated surface EMGs, but for the purpose of this study, these data were not analyzed. Data from inertial and plantar pressure sensors were recorded at 100 Hz. All the sensors were synchronized via Ephion Mobility software on a smartphone (Supplementary Figure 1). This biomechanical sensor system enables the identification of different variables, such as kinematic, foot, ankle, knee, and hip angle flexion measurements; spatiotemporal variables, such as velocity, double support time, stride length and cadence; and plantar pressure variables, such as vertical force and center of pressure (COP). Data collected from the entire test was segmented into gait cycles, which start and end when the same foot contacts the ground. A step detection algorithm using angular velocity on the sagittal plane and plantar pressure values was used to identify gait cycles in each sensor. Each gait cycle was normalized to 100 samples, representing 0 to 100% of the gait cycle, and averaged to obtain the mean pattern. This normalization facilitates the comparison of tests at different velocities. Various characteristics of the curves were then calculated from this mean cycle [43–46]. A total of six gait profiles (variables) were analyzed, including the kinematics of the hip joint, knee joint, ankle joint, and foot segment, as well as the vertical force and center of pressure evolution. Joint angles were calculated using relative segments as references, whereas the foot segment used the global reference frame. Information about which variables and features are measured with each sensor and their biomechanical interpretation are detailed in Table 1. Individuals wore the wearable system while performing the 2MWT.

**Table 1:**
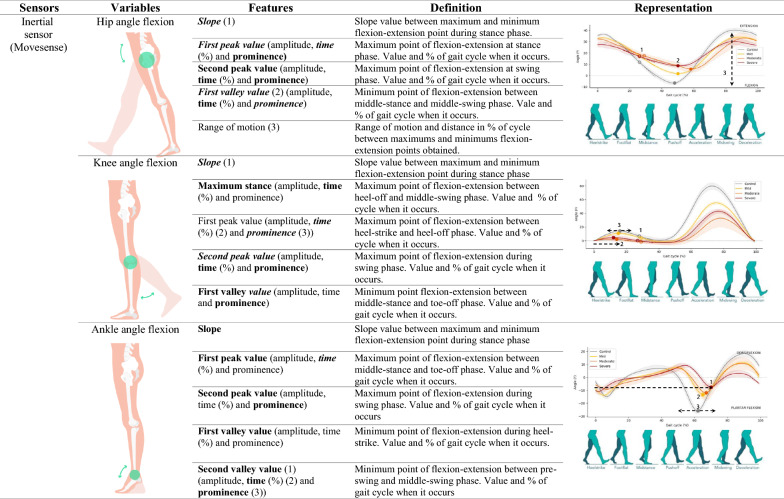

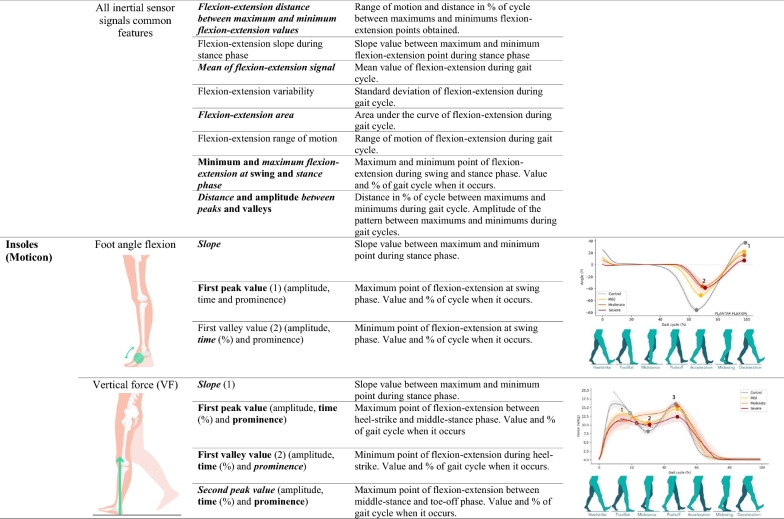

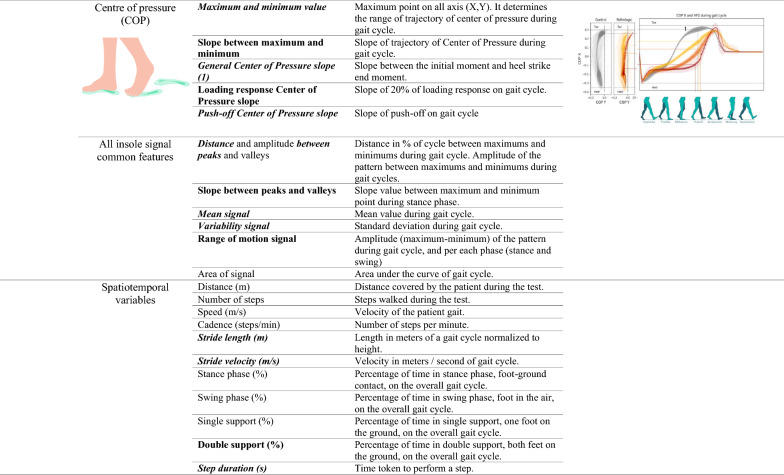
Definition and representation of variables and features measured by sensors

Sensor location and explanation of what variable and feature each sensor measures. For this table, it has been represented the curves of angular variables during a gait cycle of ataxia group of patients. Located in the representation curves, significative features obtained for the first objective; in features column in bold, significative features for the correlations study; and in features column on italic, significative features for the longitudinal analysis. Features in bold and simultaneously in italic are those significative in correlations study and in longitudinal study

### Statistical analysis

The subjects’ gait parameters were assessed by taking the mean of all gait cycles per parameter. To assess differences between the control and pathological groups (ataxia, steppage and whole cohort), Kruskal‒Wallis tests were performed. Then, multiple comparisons were performed (false discovery rate—FDR) with a corrected significance level of *p* < 0.0001 as the criterion to select features considered to effectively differentiate the patient groups from control individuals. The test statistic (H), degrees of freedom (df), and *p*-value (pFDR) for this omnibus test were recorded to evaluate the global null hypothesis of no difference between groups. Only parameters meeting this threshold were carried forward for detailed pairwise comparisons by using Tukey’s HSD test. For visualization purposes in figures, statistical significance for these pairwise comparisons is indicated using an uncorrected threshold of *p* < 0.05. We performed this analysis following previous studies [47, 48]. Features displayed in figures were selected for analytical and visual purposes. While not all displayed features reached statistical significance, they were included to illustrate representative trends across patients’ groups and support a clearer interpretation of groups differences.

Linear mixed-effects (LME) models were used to assess the relationships of the computed gait parameters with ataxia, steppage or both gait patterns. To model the relationship between clinical impact and the different gait parameters obtained in the 2MWT, we used LME using clinical scales, the iRODS and the 2MWT distance as fixed effects. A significance level of FDR-corrected *p* value < 0.05 was established to select features that effectively showed correlations for gait patterns and for clinical scales.

Finally, the Wilcoxon test and LME were used to capture clinically significant longitudinal changes in gait parameters only in patients who experienced significant clinical changes. Patients selected for these analyses included those who presented clinically meaningful worsening or improvement, defined as (1) a 2-point or greater change in the MRC score and/or (2) a 4-point change on the iRODS scale [49], which are widely accepted minimal clinically important changes in patients with PNs. For Wilcoxon analysis, the best and worst clinical assessments were selected to study the longitudinal clinical changes. For all LME analyses, participant-specific intercepts and slopes were added as random factors. A significance level of FDR-corrected *p* < 0.05 was applied to these analyses.

## Results

### Baseline clinical characteristics

For the analysis of differences in normal and abnormal gait patterns, we included 48 patients with ataxia (40 mild, 6 moderate and 2 severe), 34 with steppage (23 mild, 5 moderate and 6 severe) and 50 controls who underwent the 2MWT. Among these patients, 30 presented mixed gait patterns with ataxia plus steppage. Additionally, patients who presented normal gait patterns were excluded from this first analysis. The number of patients in each group is summarized in Supplementary Table 2. The mean age of the patients included in the ataxia group was 57.4 years, and 33 patients (68.8%) were male. The mean age of the patients included in the steppage group was 59.3 years, and 20 patients (58.8%) were male. The baseline results of the 2MWT, vigorimeter grip strength, MRCss, INCAT and iRODS scores of the included patients are summarized in Table 2 and Supplementary Table 3 for the ataxia group and in Supplementary Table 4 for the steppage group. For the analysis of the correlations between gait patterns or clinical scales and DBF, we included 79 patients with ataxia and/or steppage or normal gait patterns. For the longitudinal study, we included 31 patients who presented a change in MRCss and 22 patients who presented a change in the iRODS scale. The number of tests included in the analysis of differences between normal and abnormal gait patterns and in the longitudinal study are summarized in Supplementary Table 5.

**Table 2:** Patients’ classification according to their disease

	Grouped by disease							
	Missing	CANOMAD	CIDP	Hereditary neuropathy	IgM-MGUS	AN	Controls	*p* value
N (%)		3 (3.8)	37 (46.8)	11 (13.9)	21 (26.5)	7 (8.9)	50	
Age (years), mean (SD)	0	72.2 (9.4)	59.5 (10.4)	54.5 (15.3)	67.1 (9.3)	57.7 (21.4)	59.9 (9.4)	0.015
Sex, male (%)		2 (66.7)	20 (54.1)	4 (36.4)	19 (90.5)	6 (85.7)	17 (34.0)	
2 MWT distance (m), mean (SD)	0	109.3 (11.4)	127.1 (44.3)	117.5 (43.6)	147.0 (27.5)	127.7 (42.8)	198.0 (35.3)	< 0.001
Vigorimeter, mean (SD)								
Left	0	53.3 (25.2)	62.8 (25.6)	65.7 (25.6)	80.8 (26.4)	71.4 (20.6)	NA	
Right	0	46.0 (24.2)	61.4 (22.8)	62.2 (24.2)	79.4 (22.8)	70.6 (20.3)	NA	
MRCss total, mean (SD)	0	58.3 (1.5)	57.5 (3.0)	56.1 (3.9)	59.0 (1.8)	56.7 (3.7)	NA	NA
INCAT total, mean (SD)	1	2.3 (1.4)	2.4 (1.4)	2.4 (1.6)	1.4 (1.2)	1.9 (1.5)	NA	NA
iRODS total, mean (SD)	8	35.0 (4.2)	35.1 (7.7)	35.8 (9.3)	40.6 (5.7)	40.4 (8.3)	NA	NA

Number, mean, standard deviation (sd) of patients included of each pathology according to age and sex. Mean and sd results of 2-min-walking-test (2MWT), grip strength using vigorimeter, Medical Research Council sum score (MRCss), Inflammatory Neuropathy Cause and Treatment (INCAT), Inflammatory Rasch-built Overall Disability Scale (iRODS). NA refers to not applicable in that specific variable. Chi-quadrat test used for study the differences between sex and ANOVA test used to analyze the differences between controls and patients in 2MWT

*CANOMAD* chronic ataxic neuropathy, ophthalmoplegia, immunoglobulin M [IgM] paraprotein, cold agglutinins, and disialosyl antibodies; *CIDP* chronic inflammatory demyelinating polyneuropathy; *IgM-MGUS* monoclonal gammopathy of undetermined significance associated with IgM; *AN* autoimmune nodopathy

### DBF captures differences between normal and abnormal gait patterns

For this first analysis, statistical test results, including H values, degrees of freedom and corresponding *p*-values (pFDR) for features analyzed are summarized in Table 3.

**Table 3:** Statical results for analysis of gait patterns

	Ataxia		Steppage	
	H(3)	pFDR	H(3)	pFDR
*Spatiotemporal variables*				
Velocity	77.0	2.79e^−15^	54.9	5.12e^−11^
Stride duration	99.2	1.73e^−19^	91.3	1.32e^−17^
Normalized stride length	96.9	4.12e^−19^	73.8	9.66e^−15^
Cadence	99.2	3.41e^−20^	91.3	1.32e^−17^
Double support	72.2	1.14e^−14^	62.6	1.82e^−12^
*Vertical force*				
Slope	68.7	5.15e^−14^	51.8	1.87e^−10^
First valley value	51.5	1.47e^−11^	35.3	2.75e-7
Second peak value	21.3	0.0001	51.8	1.87e^−10^
*Center of pressure*				
Slope	60.2	2.74e^−12^	80.5	5.07e^−16^
*Foot angle flexion*				
First valley value	106.2	1.18e^−21^	79.2	8.03e^−16^
First peak value	87.7	2.19e^−17^	87.6	3.99e^−17^
*Hip angle flexion*				
Slope	87.1	1.03e^−17^	67.5	1.98e^−13^
First valley value	68.8	5.03e^−14^	54.5	5.77e^−11^
Range of motion	87.4	9.20e^−18^	54.8	5.17e^−11^
*Ankle angle flexion*				
% cycle of second valley	59.2	4.14e^−12^	48.2	8.59e^−10^
Second valley value	46.3	3.94e^−13^	55.0	4.97e^−11^
Prominence on second valley	48.5	6.06e^−10^	39.5	4.22e^−10^
*Knee angle flexion*				
Prominence on first peak	34.5	3.58e^−7^	18.6	0.0005
Slope	48.4	6.15e^−10^	22.9	8.02e^−5^
% cycle of first peak	28.4	6.15e^−6^	19.6	0.0003

**Fig. 2:**
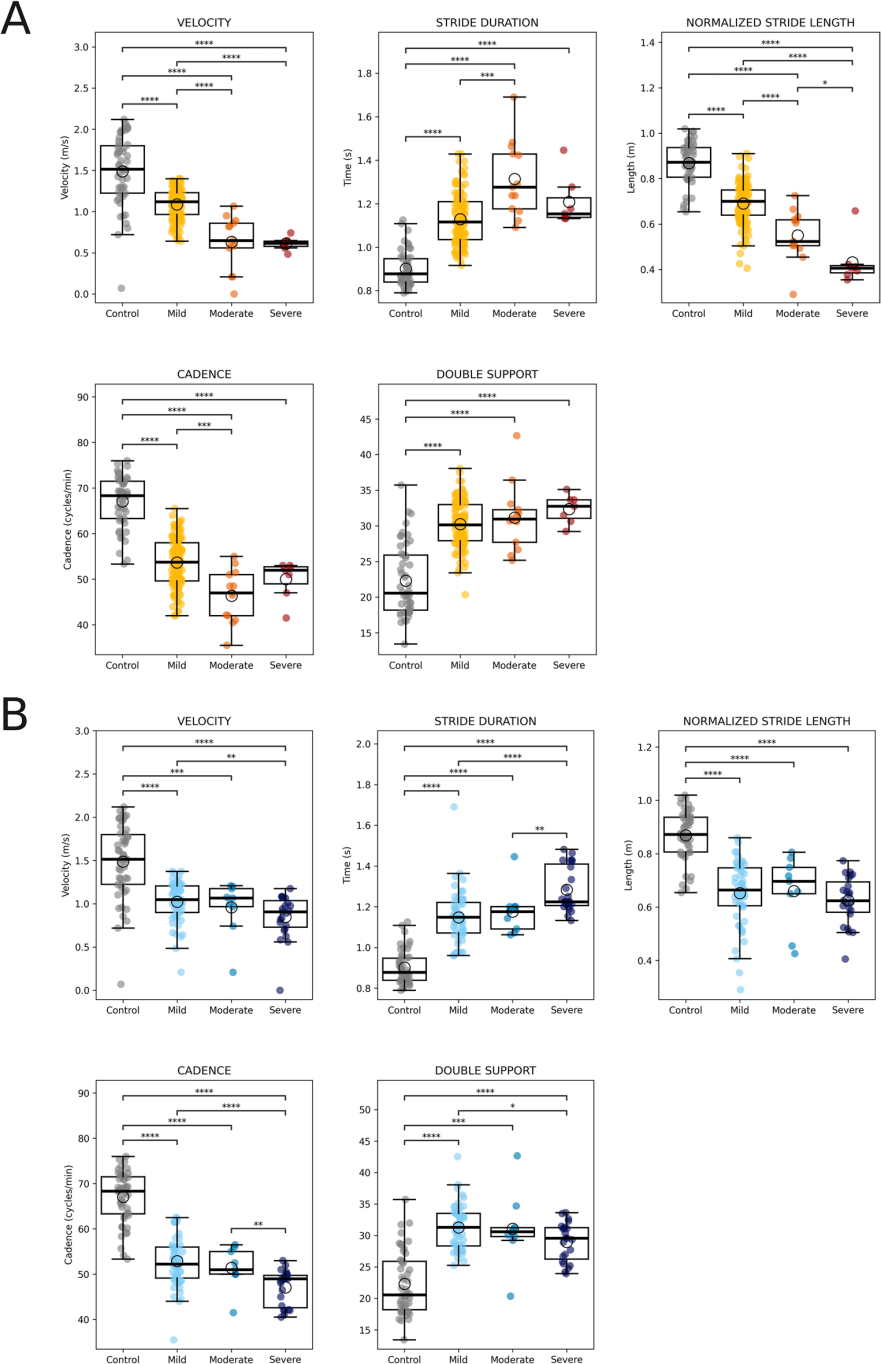
Analysis of spatiotemporal features during one gait cycle. Velocity, stride duration, double support time, stride length, cadence and stride velocity are presented for the ataxia group (**A**) and steppage group (**B**). The Kruskal‒Wallis test was performed, followed by post hoc comparisons via Tukey's HSD test. The pairwaise comparisons of the four groups are represented as follows: **** → puncorrected < 0.0001, *** → puncorrected < 0.001, ** → puncorrected < 0.01, * → puncorrected << 0.05

The spatiotemporal features (ST) velocity, stride duration and length, cadence, and double support time were analyzed for both groups of patients (Fig. 2). In patients with ataxia (Fig. 2A), there were significant overall differences between severity groups for each parameter, as indicated by the Kruskal–Wallis tests. As shown in the post-hoc comparisons in the Fig. 2A, velocity, stride length, and cadence all showed significant decreases when ataxia severity increased, while stride duration and double support time increased correspondingly. These trends were confirmed by post-hoc pairwise comparisons, as visualized in Fig. 2A. A similar pattern was observed in the steppage cohort (Fig. 2B) with significant differences in the overall and with similar trends as those observed in the ataxia group.

**Fig. 3:**
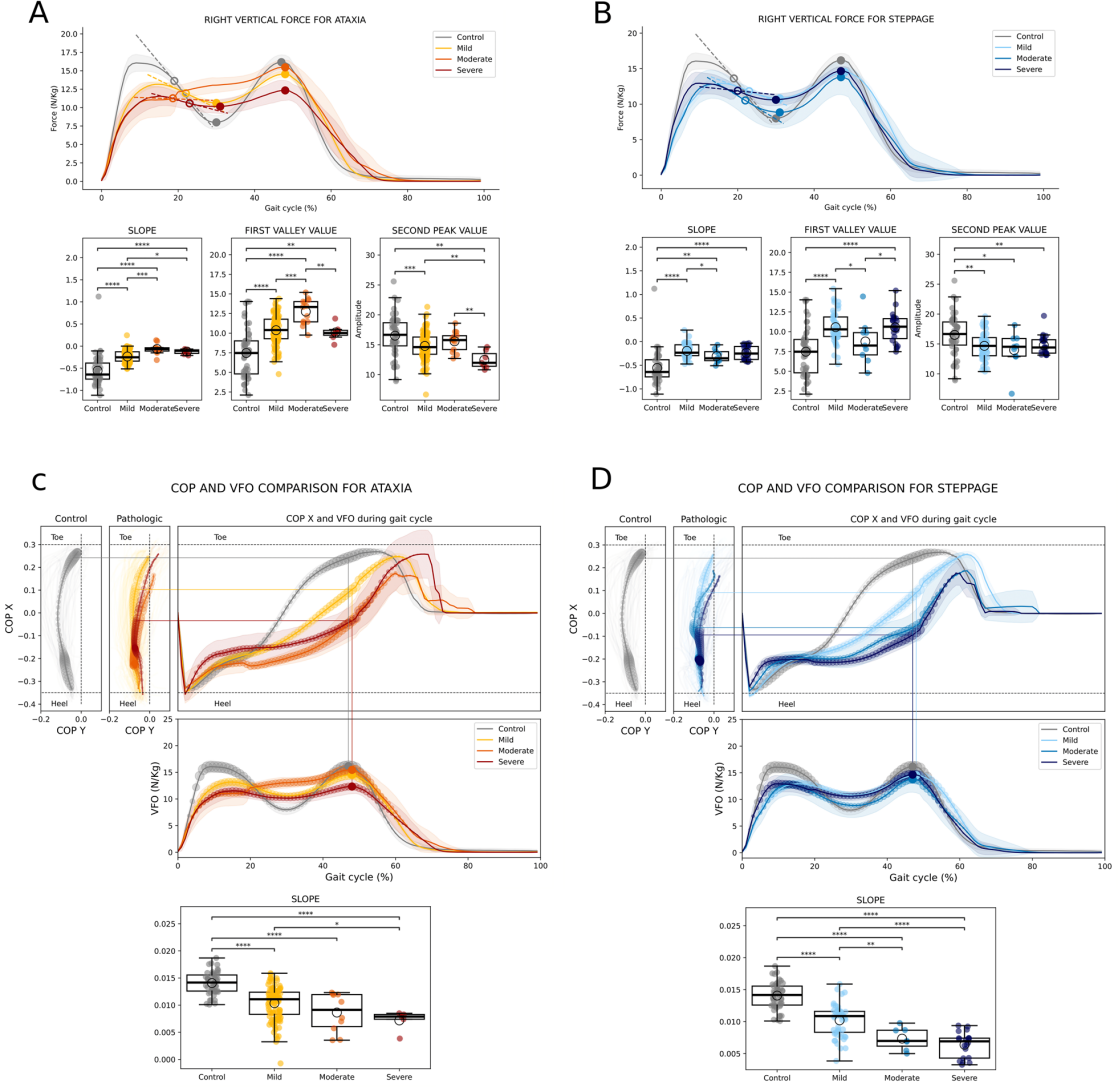
Results of the vertical force (VF) and center of pressure (COP) during one gait cycle. Representative VF for the ataxia group (**A**) and for the steppage group (**B**). Highlighted are the slope, valley value and second peak feature value. Represented COP for ataxia (**C**) and for the steppage group (**D**). The size of the bubbles appearing on the curves represents the intensity of the vertical force during each moment of the gait cycle. For both groups of patients, the slope feature was highlighted. The Kruskal–Wallis test was performed, followed by post hoc comparisons via Tukey's HSD test. The pairwaise comparisons of the four groups uncorrected differences between groupspatients and controls are represented as follows: **** → puncorrected < 0.0001, *** → puncorrected < 0.001, ** → puncorrected < 0.01, * → puncorrected < 0.05

Vertical force (VF) was analyzed throughout a gait cycle (Fig. 3A, B). For visual and analytical purposes, 3 features of the cycle were highlighted: the slope of the curve, the value in the valley, and the value of the second peak. In the ataxia group (Fig. 3A), the three gait cycle severity curves presented significantly flatter slopes compared to the control and smaller second peak as highlighted in the post-hoc comparisons. For the steppage group (Fig. 3B), differences in slopes were also significantly flatter in comparison to the control group with higher valley values and a reduced second peak.

The center of pressure (COP) of the vertical force over the insole during a gait cycle is displayed in Fig. 3C and D. Differences in slope and toe-off time were observed for both ataxia and steppage groups. A reduction of this feature was detected across the different severity groups (except for the moderate and severe groups in ataxia) as shown in the post-hoc comparisons of the Fig. 3C and D. In normal controls, the weight is distributed mainly between the heel, at the beginning of the cycle, and the toe, at the end of the cycle. In ataxia patients (Fig. 3C), the weight was distributed on the heel and along the foot during the gait cycle, and there was almost no support of the weight with the toe. Similar findings were observed for steppage patients (Fig. 3D), although differences across the severity groups were clearer. In patients with severe steppage, support almost exclusively happened on the heel. In addition, the value of the slope decreased significantly in the different severity groups.

**Fig. 4:**
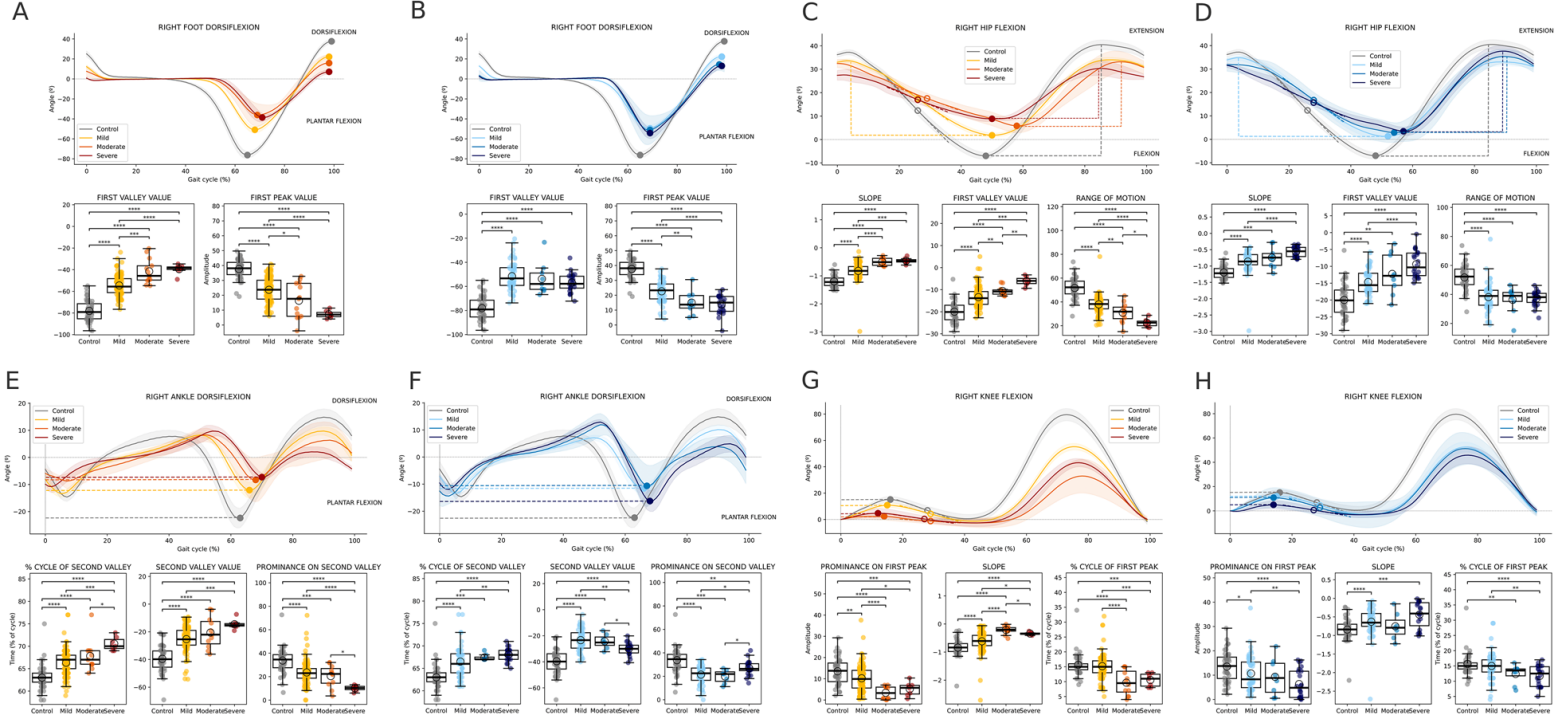
Results of foot, hip, ankle, and knee angle flexion variables during one gait cycle. (**A, B**) For the foot angle flexion variable, the value of the valley and value of the peak are highlighted. (**C, D**) For hip angle flexion, the slope, value of the valley and range motion features are highlighted. (**E, F**) For the ankle angle flexion variable, the % cycle of the second valley, the value of the second valley and the prominence of the second valleys in the discontinued line are highlighted. (**G, H**) For knee angle flexion, prominence on the first peak, slope and % cycle of the first peak in the discontinued line were highlighted. Graphics **A**, **C**, **E** and **F** are from the ataxia group, and graphics **B**, **D**, **F**, and **H** are from the steppage group. The Kruskal‒Wallis test was performed, followed by post hoc comparisons via Tukey's HSD test. The pairwaise comparisons of the four groups uncorrected differences between groups of patients and controls are represented as follows: **** → puncorrected < 0.0001, *** → puncorrected < 0.001, ** → puncorrected < 0.01, * → puncorrected < 0.05

We then analyzed foot angle flexion (Fig. 4A, B) and chose the value in the valley and the value at the second peak features for statistical comparisons. Significant global differences were found for both features in both ataxia and steppage groups. As highlighted by the post-hoc analysis, patients with ataxia (Fig. 4A) presented a significant decrease in negative foot angle flexion across the different severity groups compared with the control group during the double support phase, represented by the valley value. The same occurred during the final phase of the cycle, which corresponded to the value in the second peak, in which we observed a significant decrease in positive foot angle flexion in the different severity groups compared with the control group. Compared to the control group, the angle of flexion of the negative foot angle decreased during the double support phase, and the angle of flexion of the positive foot angle also decreased during the final acceleration phase in the group of steppage patients (Fig. 4B). However, the differences between the severity groups were smaller than those between the ataxia groups.

Significant global differences were found for all three features in the hip angle flexion (Fig. 4C, D) in both ataxia and steppage groups. The post-hoc comparison revealed a decreased slope feature and a significantly lower negative hip angle across the different severity groups of ataxia patients (Fig. 4C). The range of motion feature was also significantly lower in the different severity groups. For the steppage group (Fig. 4D), significant differences were observed in the value of the slope for the three severity groups compared with the control group. The values at the valley and the range of motion significantly changed only when patients and controls were compared but not across the three severity groups.

The curves of the ankle flexion variable (Fig. 4E, F) were significantly different in both ataxia and steppage groups. Post-hoc analysis revealed a delay in the appearance of the valley in patients with ataxia (Fig. 4E). A decrease was also observed in the negative value of the valley feature across the different severity groups compared with the control group. For this variable, the proportion of the cycle in the second valley significantly decreased across the different severity groups. The opposite occurred for the prominence feature, which increased significantly across the severity groups. For steppage patients (Fig. 4F), the same findings were observed: a delay in the appearance of the valley, a decrease in the negative value of the valley and in the % cycle of the second valley, and an increase in the prominence feature. The differences across severity groups were less prominent than those in the ataxia group.

For the knee angle flexion variable (Fig. 4G, H), significant differences were found for the ataxia and steppage group of patients. Figure 4G showed a flattening of the entire first phase of the gait cycle observed in patients with ataxia, with a significant decreased in the slope (close to 0 across the different severity groups). The prominence and proportion of first peak features significantly decreased (except for those in the severe group) across the different severity groups. For the steppage group of patients (Fig. 4H), the same trends were observed: a decrease in the negative slope and a decrease in the prominence and proportion of first peak features. For the steppage group, significant differences were obtained only when patients and controls were compared and not across all severity groups.

### DBF correlates with gait patterns in the global population of patients with PNs

After the ability of the DBF system to capture deviations from normality in the different features was studied, the relationships of these features with their gait patterns were analyzed. The same cohort of patients was selected, and the LME test was used to analyze which biomechanical features correlated with each gait phenotype.

**Fig. 5:**
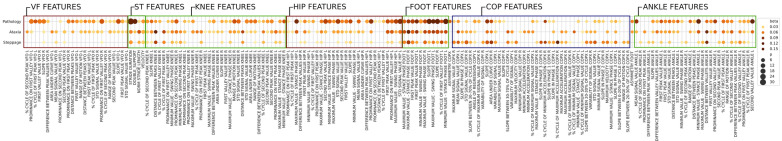
Correlation of gait patterns with biomechanical features. Color intensity and size of the bubbles represent the correlation level of the features with their gait pattern. Boxed in red, features related to ataxia gait pattern; boxed in blue, features related to steppage gait and boxed in green, features related to both gait patterns. Multiple comparisons were performed and a significance level of FDR-corrected *p*-value < 0.05 was established to select features that correlated with their gait pattern. Only plotted significant features. VF: Vertical force; ST: Spatiotemporal; COP: Center of pressure

LME analysis revealed 219 biomechanical features associated with ataxia, steppage or both gait patterns (Fig. 5). Among these features, 131 features were associated with ataxia, 94 with steppage, and 133 with both gait patterns. Among these features, 24 were associated only with ataxia, and 10 were associated only with steppage. Interestingly, while the features associated with only gait ataxia were related mainly to plantar pressure and hip features, those correlated with steppage were related mainly to COP features. As previously observed in Fig. 3C and D, significant differences in COP features were observed among steppage patients and across the severity groups. Biomechanical features related to the foot, knee, ankle, and spatiotemporal variables correlated with both gait ataxia and steppage gait, in line with previous analyses shown in Figs. 2 and 4.

### DBF correlates with conventional impairment and disability clinical scales in the global population of patients with PNs

**Fig. 6:**
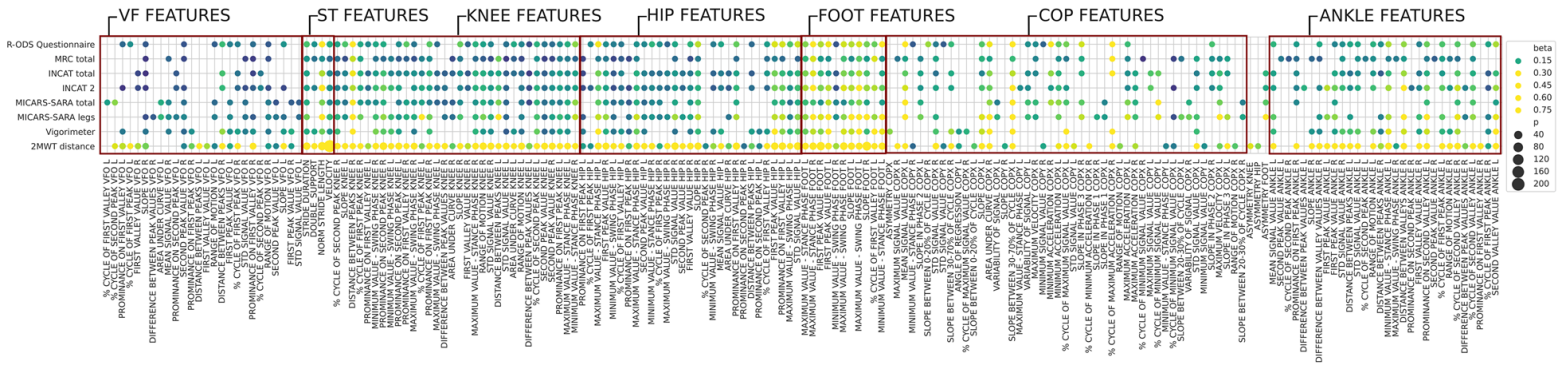
Correlation of clinical scales with biomechanical features. Color intensity and size of the bubbles represent the correlation level of the features with their gait pattern. Non-normalized and normalized scales are represented using the logarithm of the total score. Multiple comparisons were performed and a significance level of FDR-corrected *p*-value < 0.05 was established to select features that correlated with their gait pattern. Only plotted significant features. VF: Vertical force; ST: spatiotemporal; COP: Center of pressure; MRCss: Medical Research Council sum score, INCAT: Inflammatory Neuropathy Cause and Treatment, iRODS: Inflammatory Rasch-built Overall Disability Scale, MICARS-SARA: Modified Internacional Cooperative Ataxia Rating Scale and Scale for the Assessment and Rating of Ataxia, 2MWT: 2-min walking test

For this analysis, the full cohort of patients was included, and the LME test was used to analyze which biomechanical features correlated with the different clinical scales (Fig. 6).

A total of 219 features were extracted, and 182 of them correlated significantly with any of the impairment (MRCss, MICARS-SARA, and vigorimeter) or disability (INCAT and iRODS) clinical scales and the 2MWT. Four ST features, eleven foot features, ten hip features, and seventeen knee features were strongly correlated with all the clinical scales and the 2MWT. Thirty-seven (17%) features did not correlate with any clinical scale. Other features related to the VF, COP, hip and ankle correlated with some but not all the scales, and 19 (8.7%) of them correlated with only one clinical scale or 2MWT. Focusing on the scales, iRODS correlated with 112 features (51.1%), MRCss with 100 features (45.7%), INCAT with 115 features (52.5%), MICARS with 96 features (43.8%), vigorimeter with 95 features (43.4%) and 2MWT with 147 features (67.1%) related to some of the VF, COP, knee, hip, and ankle features.

### DBF captures longitudinal changes in patients with neuropathies

We then studied the ability of the DBF to capture longitudinal changes in patients with PNs. Patients selected for these analyses included those who presented clinically meaningful worsening or improvement, defined as (1) a 2-point or greater change in the MRC score and/or (2) a 4-point change on the iRODS scale, which are conventional definitions for minimal clinically important differences in PNs. We detected 41 CIDP patients (119 tests), 20 IgM-MGUS-associated neuropathies (54 tests), and 11 hereditary neuropathies (16 tests) that fulfilled these criteria and compared their results with those of a longitudinal assessment of 50 healthy controls.

**Fig. 7:**
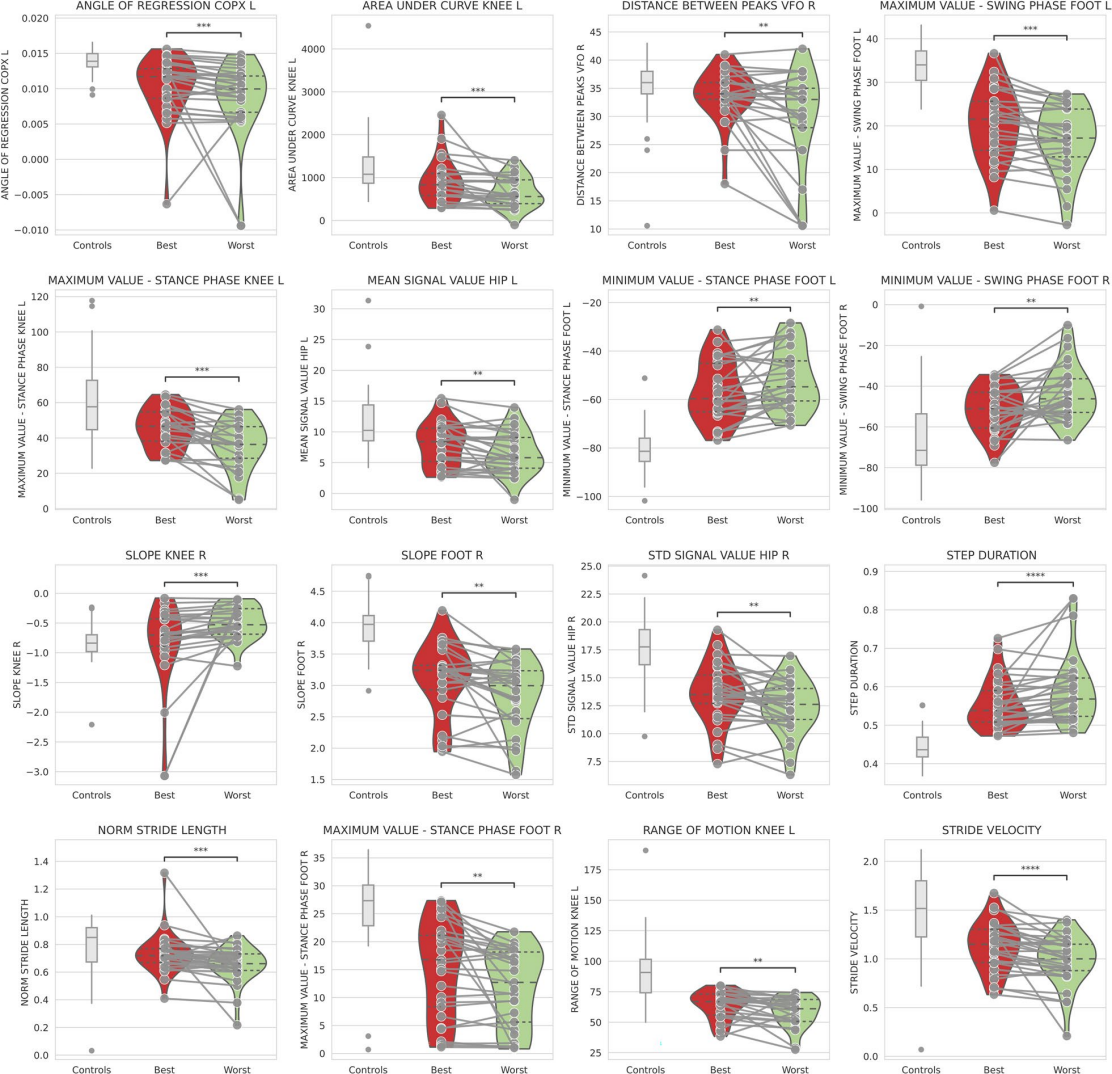
Features that detected clinically significant changes in longitudinal analysis via the Wilcoxon model. The best and worst clinical assessments were selected in a cohort of patients who showed a difference of at least 2 points on the MRCss. Multiple comparisons were used, and a FDR-corrected *p* value < 0.05 was established to differentiate the features that significantly differed over the time. MRCss: Medical Research Council sum score

When the best and worst clinical assessments in patients who showed a significant change in the MRCss scale were selected, significant differences were observed in 16 biomechanical features (Fig. 7), mainly those related to foot biomechanics. Additionally, differences were observed for COP variables and stride length features. Using the same statistical test but with a cohort of patients who presented a change of at least 4 points on the iRODS scale, we did not detect significant changes in any biomechanical feature, probably because the iRODS, a subjective scale, may fluctuate in the absence of objective change.

**Fig. 8:**
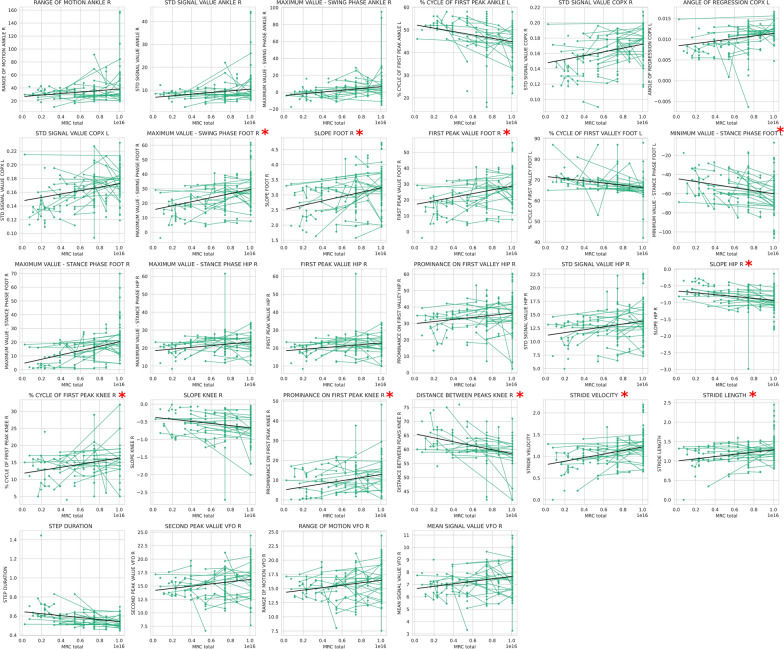
Features that detected clinically significant changes in longitudinal analysis via the LME model. All available longitudinal assessments were used in a cohort of patients who showed a clinical change of 2 points on the MRCss scale. Multiple comparisons were used, and a FDR-corrected *p* value < 0.05 was established to differentiate the features that significantly differed over time. *Features that were also present in the cohort of patients who presented a clinical worsening on the iRODS scale. LME: linear mixed effects, MRCss: Medical Research Council sum score, INCAT: Inflammatory Neuropathy Cause and Treatment

**Fig. 9:**
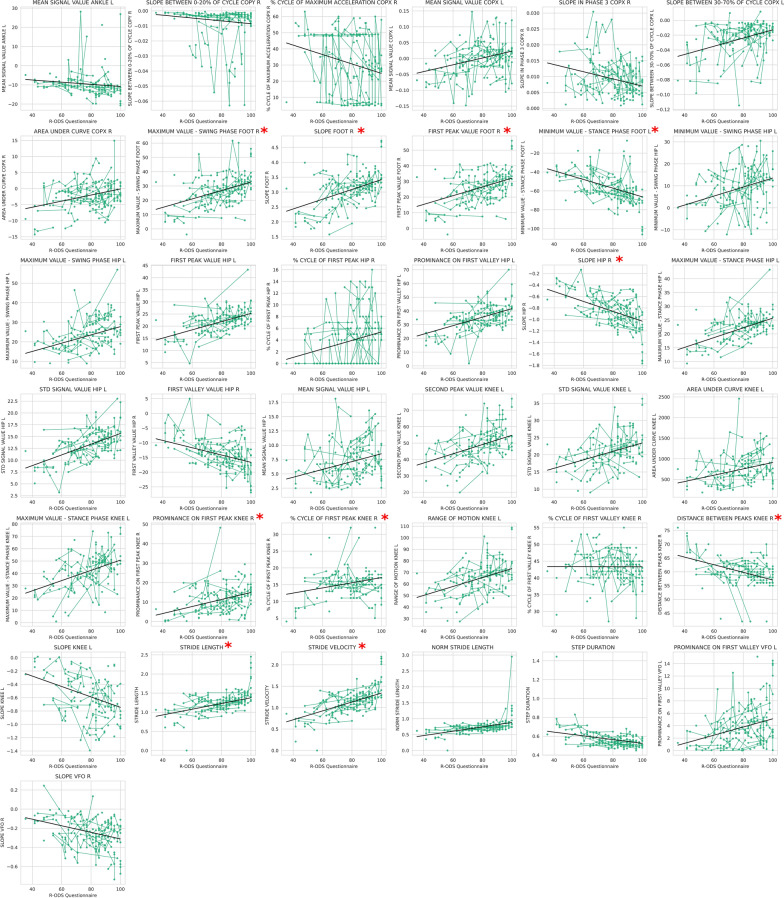
Features that detected clinically significant changes in longitudinal analysis via the LME model. All available longitudinal assessments were performed in a cohort of patients who experienced a clinical change of 4 points or more on the iRODS scale. Multiple comparisons were used, and a FDR-corrected *p* value < 0.05 was established to differentiate the features that significantly differed over the time. *Features that were also present in the cohort of patients who presented a clinical worsening in the MRCss scale. LME: linear mixed effects, MRCss: Medical Research Council sum score, iRODS: Inflammatory Rasch-built Overall Disability Scale

When all available longitudinal assessments with LME analysis were used in a cohort of patients who presented a clinically relevant change in the MRCss at any time point, the DBF system was able to identify 28 different biomechanical features correlated with clinical changes (Fig. 8), which were related to the foot, COP, ankle, and velocity. Finally, in a cohort of patients who had a change of 4 points in the iRODS, 37 biomechanical features correlated with the clinical change according to the same LME statistical test (Fig. 9). The features capturing the clinically relevant changes were those related to the foot. Significant longitudinal differences were also detected in features related to the hip and diverse spatiotemporal features in this cohort of patients. Ten of these features, four of which were related to foot function, detected the clinical changes classified by both the iRODS and MRCss (Figs. 8 and 9, marked in red*). Additionally, three of these features were related to knee function, two were related to spatiotemporal variables, and one was related to hip function.

## Discussion

Our study provides proof of concept that DBF capture gait impairments in patients with PNs offering a less subjective approach than conventional clinical methods and showing responsiveness to changes in clinical status. DBF alterations correlate with PN patient impairment and disability, as measured with clinical scores. Moreover, longitudinal analysis of specific DBF features revealed clinical changes in patients with PNs. This suggests that, if validated in appropriate clinical trials, DBF could be used to monitor disease status and capture and quantify longitudinal changes more objectively than clinical scores in patients with PNs. These quantifiable assessments are achieved by integrating data signals captured with easy-to-use multiparametric sensors that can be simplified or tailored to select the most informative features in each type of gait phenotype.

A critical unmet need in the field of peripheral neuropathies is the absence of more objective diagnostic, prognostic, and disease activity biomarkers [13]. Current monitoring strategies are based on clinical examinations and scores that are affected by subjective interpretation, imprecision, nonrelevant fluctuations and ceiling and floor effects. Ultimately, this results in substantial uncertainty regarding the clinical status of patients, particularly those with predominantly subjective symptoms, or those in which residual disability, treatment fluctuations and treatment side effects interfere with the proper evaluation of their clinical status [50]. Moreover, the subtle nature of clinical changes in hereditary neuropathies and the important placebo effect and the subjective and objective clinical fluctuations in patients with inflammatory neuropathies also influence the ability of clinical trials to detect significant changes, since the magnitude of the effect needs to overcome the substantial statistical noise that these imprecise scales provide. For these reasons, our main objective was to evaluate whether gait alterations (one of the main clinical manifestations in patients with PN) could be quantitatively assessed via a novel, multiparametric DBF that is able to capture and quantify the biomechanical alterations associated with gait impairment.

We first proved that DBF is able to detect differences in two different gait phenotypes commonly observed in patients with PNs (ataxia and steppage gait) and across severity groups. Second, we demonstrated that some DBF alterations correlate with conventional clinical scales and PROMS. Finally, we validated the ability of DBF to detect changes in the different features over time.

Gait impairment is one of the most characteristic alterations in patients with PNs [33, 37]. As such, one of the most obvious alterations in the clinical assessment of gait is a decrease in gait speed. For this reason, timed walking tests are popular in the assessment of PNs and other neuromuscular disorders [23]. However, the exact parameters that determine a reduction in gait speed differ across patients, and there may be subtle alterations in specific parameters even if, globally, gait is not visually impaired or if the distance achieved during the timed test is considered within normal ranges. Dinesh et al. [31] focused on the utility of wearable sensors in patients with Charcot-Marie-Tooth disease type 1A (CMT1A), correlating spatiotemporal gait and balance metrics with clinical scores. While their work established foundational tools for tracking motor deficits in a specific hereditary neuropathy, our study extends this approach in several important ways. First, our study includes a broader range of PNs, enabling more generalizable findings. Second, we focused on a larger and more diverse feature set including vertical ground reaction force parameters and lower-limb kinematics which are particularly sensitive to changes in balance and motor control. Third, our study emphasizes longitudinal tracking and the potential of DBF to detect subclinical change. These aspects enhance sensitivity to subtle motor changes and support broader clinical applicability for heterogeneous PN populations.

Considering the frequent alteration of gait speed in patients with PNs, we consequently observed that spatiotemporal parameters related to gait speed, such as velocity, stride length and cadence, decreased significantly in these patients; in addition, the parameters related to time increased significantly with severity. Although these alterations in spatiotemporal gait features are detected in both ataxia and steppage phenotypes and strongly correlate with all clinical scores, the specific features that are altered and the pattern of abnormality differ depending on the type of gait. Essentially, patients with ataxia or steppage walk slower than normal controls do, but the features behind this decrease in speed are different in each gait phenotype, and the degree of alteration of each spatiotemporal feature varies depending on the severity of the disease.

Similarly, gait impairment in patients with PNs also results in footprint abnormalities that vary depending on gait phenotype. Consequently, alterations in plantar pressure or vertical force allowed us to distinguish both groups of patients whose gait cycle curves were significantly different from each other. This parameter clearly captured significant differences across the different levels of severity, especially in the ataxia group. In the severe ataxia group of patients, the inversion of the second peak was lost because these patients presented high gait instability, and both feet barely lifted off the ground. However, the double support phase was not impaired to the same extent in steppage patients. Footprint abnormalities are visually obvious in patients with gait alterations, but our study again provides objective and quantifiable evidence of these alterations and detects which features are specific for each type of gait.

In steppage patients, the main gait alteration is foot drop. This finding suggests that plantar pressure is likely significantly disrupted in patients whose foot control is significantly impaired. As such, COP features (in which the plantar distribution of pressure that the foot exerts when touching the ground is evaluated) were the only features that showed a significant correlation with the steppage gait pattern. COP features on the steppage were significantly disrupted and differed among the three severity groups compared with those of the control group. These results mirror what is observed in the clinic, where patients whose foot and toe drop do not exert plantar pressure in the anterior regions of the insole pressure detectors. However, although differences were observed between ataxia patients and controls, there were no differences across severity groups.

The foot, hip, ankle, and knee angle flexions also exhibited notable deviations from normal in the different patient groups. Patients with ataxia show a pattern of gait that results from the presence of imbalance, whereas steppage gait appears due to the presence of distal leg weakness. Since steppage gait abnormalities are more focally distributed, deviations from the normality of leg angle features of the DBF were more generalized and profound in patients with ataxia. For these reasons, foot flexion angle features were altered in both groups of patients, whereas hip angle flexion appeared flattened and correlated with severity in the ataxia group only. Patients with steppage do not show flat curves in hip flexion because, when their foot is dropped, they exert additional force from the hip to be able to lift the entire foot, preserving or even exaggerating the width of the hip angle. In contrast, ataxic patients tend to walk without bending their hips and knees or widening the sustentation base to increase stability and avoid falls. Ankle and knee flexion feature alterations were more profound, as were other angle flexion features, for ataxia patients. Leg joint angle features made it possible to stratify the different severity groups in ataxia patients but not in steppage patients, in which differences were only able to distinguish patients from controls.

While correlation with validated clinical scales may support interpretability, DBF may be able to anticipate changes in clinical scores or provide complementary dimensions of patients’ function that may not be captured by conventional assessments. Nevertheless, in the group of diseased patients, the 2MWT and all impairment and disability clinical scales had strong correlations with DBF features related to the foot and with some of the temporospatial variables. The 2MWT, the test used for all data analysis, proved to be, as expected, the test that correlated with more biomechanical features. Some biomechanical features did not show correlations for any clinical scale or for the 2MWT. Grip strength, which was assessed with a vigorimeter, and the iRODS, a disability scale validated to capture the full range of disability in inflammatory neuropathies, also showed significant correlations with vertical force features; COP features; and ankle, hip and knee angles . Some of these correlations may be obvious and visually detected since increased severity of a neuropathy will certainly correlate with spatiotemporal features. However, this exhaustive analysis helps uncover other, less obvious correlations, such as those between grip strength and COP features. Overall, these results indicate that biomechanical alterations correlate with the clinical status of patients and, therefore, could offer a quantifiable and less subjective way to characterize deviations from normality.

To be clinically useful for monitoring disease in clinical practice or in clinical trials, DBF needs to be able to capture longitudinal changes in addition to correlating with clinical scales and capturing different grades of severity. To initially test the ability of DBF to capture longitudinal changes, we selected patients for whom a clinically significant change was defined by 2 points on the MRCss scale or 4 points on the iRODS scale, which are two frequently used criteria to detect a minimal clinically important difference and analyzed their DBF features. Several DBF features were able to detect longitudinal changes over time: when all available data were used, 28 and 37 features detected clinical changes defined with MRCss and iRODS, respectively. Most of them are related to foot features because, when weakness appears in patients with PNs, it most frequently starts in the most distal muscles. However, if, instead of considering all available tests, we selected the best and worst clinical assessments in these two cohorts of patients, 16 DBF features became significant for the MRC group, suggesting that DBF monitoring is not only able to detect minimal clinical longitudinal changes but also that when the disease progresses further, as expected, other DBF features become sensitive to those changes. These results demonstrate that the DBF features most sensitive for detecting longitudinal changes are those related to the foot. This can certainly have implications for setting the minimum number of parameters that are clinically useful so that gait analysis can be simplified. There were no significant changes in any biomechanical features when we selected the best and worst clinical assessments and the clinical status was stratified with the iRODS. This is likely because the changes detected by the iRODS scale are more subtle and influenced by the patient's subjectivity and due the fact that iRODS is optimized for autoimmune neuropathies and not for any type of neuropathy.

Nonetheless, this longitudinal analysis is subject to limitations. While it provides valuable insight into the evolution of gait patterns in PN patients, we recognize that certain clinical fluctuations remain beyond resolution of our measurement framework. Fatigue or transient treatment effects can introduce variability not captured at fixed assessment points and this variability can undermine the precision of our analysis, especially when attempting to detect clinically meaningful change. Although the thresholds used in our analysis are commonly used to define clinical deterioration, as pointed out by Veen et al. [51], even these established cut-offs may fall within the natural variability of stable patients, limiting their sensitivity and specificity at the individual level, making it challenging to distinguish true deterioration from background noise, and influencing the ability of DBF changes to correlate with clinical changes. Thus, defining DBF-specific minimally clinically important changes independently to clinical scores is essential to properly understand the utility of DBF in the assessment of gait changes. Performing such analyses was beyond the scope this proof-of-principle study.

There are several limitations for multiparametric wearable technology that need to be acknowledged. From a feasibility point of view, the duration of visits that incorporate DBF analysis is significantly longer than that of conventional visits since all sensors need to be placed and synchronized. This limitation could be mitigated by choosing a set of sensors that provides sufficient information to capture clinically relevant alterations across groups and longitudinally within groups. Another option is to restrict the use of the technology to research settings, for example, in clinical trials, in which additional time can be allocated to use these technologies. Additionally, handling and interpreting massive amounts of biomechanical data is challenging. In the future, the simplification of the protocol, linked to the development of clinically validated software that simplifies the interpretation of the findings, would be necessary to incorporate the technology into the clinical routine.

Our study also has several limitations. First, and most importantly, a diverse set of patients with neuropathy, with different clinical features, different severities, and different longitudinal behaviors, is incorporated. Although we believe that this approach is interesting if we want to find biomechanical features that are useful for monitoring any PN patient, regardless of type, focused studies stratifying each disease type could provide more precise DBF features to be used in each neuropathy subtype. Nevertheless, the fact that we were able to capture DBF features correlated with clinical status and longitudinal changes despite the heterogeneity of the population suggests that a disease-focused approach would further increase the sensitivity of this technology. Future analyses should consider stratification of findings according to disease instead of stratification according to gait phenotype. Another limitation of the study is that our DBF protocol is most useful when gait impairment is present. Although this is a very frequent feature in patients with neuropathy, it is not universal. Future studies should address whether this technology is also able to capture minor DBF alterations in patients whose gait disturbances are not obvious. Another limitation of the study is that these results are applicable only to adult populations. How DBF alterations behave in children with neuropathies and if those are comparable to findings in adults is a pending task. Finally, all these results arise from the interpretation of individual DBF feature data. An important step is to develop an analytical framework in which the interplay between diverse DBF features and clinical scores can provide a more precise picture of the clinical situation of a patient.

Our findings challenge the current paradigm that relies on conventional clinical scales as the primary reference for assessing clinical status in PN patients. Although these scales are widely used, they are limited by subjectivity, ceiling effects, and insensitivity to subtle changes, issues that our multiparametric wearable approach aims to address. Results obtained in this study highlight the potential of biomechanical features to provide a more precise understanding of motor function. However, further studies are needed to validate this approach exploring dynamic and static assessments of the upper limb and postural control, extending beyond gait to build a more comprehensive, sensor-based framework for monitoring disease activity.

## Conclusions

Our study provides proof-of-concept that noninvasive wearable biomechanical sensors are useful for capturing, quantifying and monitoring gait disturbances in patients with PNs in an objective and reproducible way. Future multicenter clinical trials should address if DBF is reproducible, if stratification according to disease type improves its performance and if it is able to detect subtle alterations in patients in which gait disturbances are not visually obvious. Successful validation of the DBF system will help overcome the limitations of current monitoring strategies by providing more objective and precise monitoring in PNs, which will help optimize patient care and, ultimately, improve the quality of life of these patients.

## Supplementary Information

Additional file 1.

Additional file 2.

## Data Availability

The datasets generated and/or analyzed during the current study are available from the corresponding author upon reasonable request.

## Abbreviations

CANOMAD: Chronic ataxic neuropathy, ophthalmoplegia, immunoglobulin M paraprotein
CIDP: Chronic inflammatory demyelinating polyneuropathy
CMT1A: Charcot–Marie–Tooth disease type 1A
COP: Center of pressure
DBF: Digital biomechanical features
DF: Degrees of freedom
DPN: Diabetic peripheral neuropathy
EMG: Electromyography
FDR: False discovery rate
INCAT: Inflammatory neuropathy cause and treatment
IgM-MGUS: Monoclonal gammopathy patients of undetermined significance associated with IgM
iRODS: Inflammatory Rasch-built Overall Disability Scale
LME: Linear mixed-effects
MICARS-SARA: Modified internacional cooperative Ataxia Rating Scale and Scale for the Assessment and Rating of Ataxia
MRCss: Medical Research Council sum score
PNs: Peripheral neuropathies
ST: Spatiotemporal
VF: Vertical force
2MWT: 2-Minute walking test
