# Supplementary material for: Digital biomechanical assessment of gait in patients with peripheral neuropathies
Source: J Neuroeng Rehabil. 2025 Jul 13;22:159. doi: 10.1186/s12984-025-01694-w (PMC12257721; doi:10.1186/s12984-025-01694-w)
Supplement: Supplementary file 1 — Additional file 1. [file 12984_2025_1694_MOESM1_ESM.pdf]

# Supplementary figures

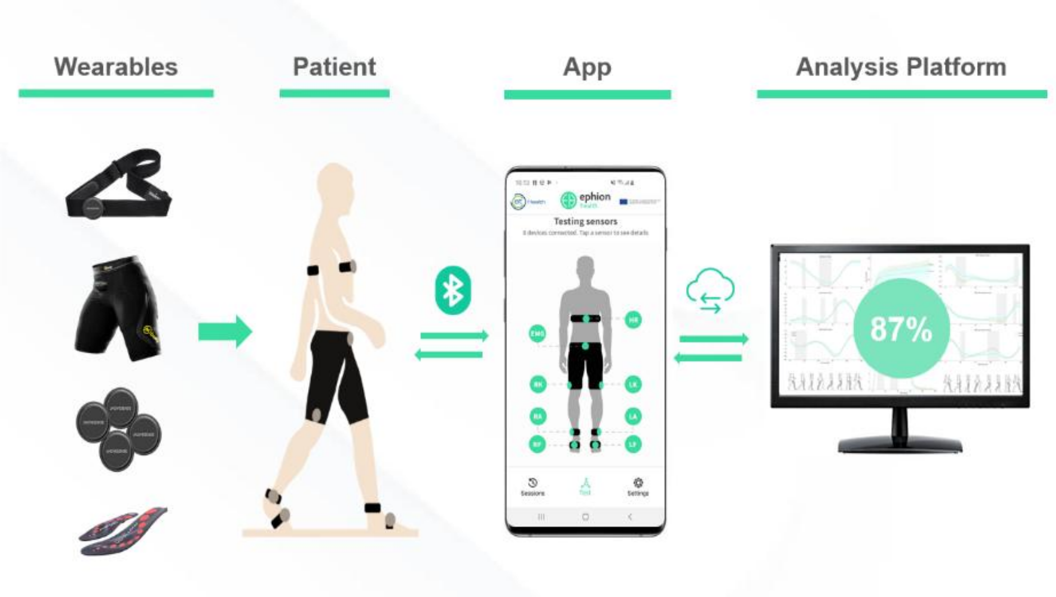


**Supplementary Fig. 1 Ephion Mobility System.** It is made up of 5 inertial sensors located in different parts of the body, pants with surface EMG and insoles with inertial and plantar pressure sensors. Using Bluetooth these sensors are synchronized into smartphone and to a platform to perform the data analysis.
