# Supplementary material for: Digital biomechanical assessment of gait in patients with peripheral neuropathies
Source: J Neuroeng Rehabil. 2025 Jul 13;22:159. doi: 10.1186/s12984-025-01694-w (PMC12257721; doi:10.1186/s12984-025-01694-w)
Supplement: Supplementary file 2 — Additional file 2. [file 12984_2025_1694_MOESM2_ESM.pdf]

Supplementary tables

Supplementary Table 1. Gait pattern classification in ataxia or steppage and according to de severity.

	Ataxia<br>MICARS-SARA item1<br>(walking capacities)	Steppage<br>MRC-SS Ankle Dorsiflexion<br>item (Right + Left)
**Severe**	Value ≥ 5	Value ≤ 4
**Moderate**	Value 3-4	Value 5-6
**Mild**	Value 1-2	Value 7-8
**Normal**	Value 0	Value ≥ 9

Categorization performed following the score obtained in item 1 of MICARS-SARA scale for ataxia patients and following the score of dorsiflexion item for steppage patients. MICARS-SARA: Modified Internacional Cooperative Ataxia Rating Scale and Scale for the Assessment and Rating of Ataxia; MRCss: Medical Research Council sum score.

Supplementary Table 2. Patients included in the study according to their gait pattern.

	Gait Pattern		N
	**Steppage**	**Ataxia**	
	Mild	Mild	17
		Moderate	2
		Severe	-
		Normal	4
	Moderate	Mild	3
		Moderate	1
		Severe	1
		Normal	-
	Severe	Mild	4
		Moderate	2
		Severe	-
		Normal	-
	Normal	Mild	16
		Moderate	1
		Severe	1
		Normal	26
**Not specified**			1

Number of patients with ataxia and/or steppage and patients with normal gait pattern. Patients with normal gait pattern were excluded for the analysis of normal and abnormal gait pattern whereas in the study of correlations with gait patterns these patients were included.

Supplementary Table 3. Baseline clinical characteristics according to the severity in ataxia group.

		Grouped by ATAXIA						
		**Missing**	**Mild**	**Moderate**	**Severe**	**Normal gait**	**Controls**	**p-value**
**N**			40	6	2	30	50	
**Age (years), mean (SD)**		0	62.3 (12.7)	67.4 (10.4)	57.4 (5.8)	58.7 (13.5)	59.9 (9.4)	0.360
**Sex, male (%)**			29 (72.5)	3 (50.0)	1 (50.0)	18 (60.0)	17 (34.0)	
**2 MWT distance (m), mean (SD)**		0	124.8 (26.2)	54.0 (40.4)	79.0 (15.6)	158.2 (28.7)	198.0 (35.3)	<0.001
**Vigorimeter, mean (SD)**	Left	50	67.8 (22.4)	45.5 (13.3)	26.0 (19.8)	77.8 (27.0)	NA	<0.002
	Right	50	66.8 (21.1)	45.7 (11.1)	54.0 (22.6)	71.9 (27.3)	NA	0.081
**MRC total, mean (SD)**		50	57.6 (2.4)	51.8 (4.2)	53.5 (3.5)	59.1 (1.4)	NA	<0.001
**INCAT total, mean (SD)**		51	2.0 (1.1)	4.2 (1.2)	5.0 (0.0)	1.6 (1.2)	NA	<0.001
**iRODS total, mean (SD)**		58	36.3 (6.4)	25.2 (6.5)	21.5 (2.1)	41.5 (5.3)	NA	<0.001

Number, mean, standard deviation (sd) of patients included of each pathology according to age and sex. Mean and sd results of 2-minute-walking-test (2MWT), grip strength using vigorimeter, Medical Research Council sum score (MRCSS), Inflammatory Neuropathy Cause and Treatment (INCAT), Inflammatory Rasch-built Overall Disability Scale (iRODS). Chi-quadrat test used for study the differences between sex. ANOVA test used to study the differences between each severity group for the clinical scales and 2MWT. A significance level of p-value <0.0001 established to differentiate these patient groups.

Supplementary Table 4. Baseline clinical characteristics according to the severity in steppage

		Grouped by STEPPAGE						
		**Missing**	**Mild**	**Moderate**	**Severe**	**Normal gait**	**Controls**	**p-value**
**N**			23	5	6	44	50	
**Age (years), mean (SD)**		0	58.4 (14.8)	60.5 (13.6)	58.9 (15.1)	63.1 (11.3)	59.9 (9.4)	0.954
**Sex, male (%)**			14 (60.9)	3 (60.0)	3 (50.0)	31 (70.5)	17 (34.0)	
**2 MWT distance (m), mean (SD)**		0	116.8 (33.0)	99.0 (41.1)	85.8 (50.0)	148.2 (32.5)	198.0 (35.3)	<0.001
**Vigorimeter, mean (SD)**	Left	50	64.3 (22.0)	55.8 (30.9)	55.0 (27.1)	74.6 (26.1)	NA	0.114
	Right	50	62.5 (20.6)	60.4 (15.3)	57.0 (28.4)	71.2 (25.3)	NA	0.312
**MRC total, mean (SD)**		50	56.9 (2.0)	53.8 (3.0)	51.5 (3.5)	59.3 (1.3)	NA	<0.001
**INCAT total, mean (SD)**		51	2.4 (1.5)	3.5 (1.3)	2.7 (1.6)	1.7 (1.2)	NA	0.023
**iRODS total, mean (SD)**		58	34.8 (8.0)	29.8 (11.3)	35.5 (7.2)	39.2 (6.6)	NA	0.028

Number, mean, standard deviation (sd) of patients included of each pathology according to age and sex. Mean and sd results of 2-minute-walking-test (2MWT), grip strength using vigorimeter, Medical Research Council sum score (MRCSS), Inflammatory Neuropathy Cause and Treatment (INCAT), Inflammatory Rasch-built Overall Disability Scale (iRODS). Chi-quadrat test used for study the differences between sex. ANOVA test used to study the differences between each severity group for the clinical scales and 2MWT. A significance level of p-value <0.0001 established to differentiate these patient group.

Supplementary Table 5. Number of tests included in the analysis of the differences between normal and abnormal gait pattern and in the longitudinal analysis.

Analysis of differences of normal and abnormal gait patterns							
**Gait pattern**	**Level**	**CANOMAD**	**CIDP**	**Hereditary neuropathy**	**IgM-MGUS**	**AN**	**N Total**
**Control**	**-**	-	-	-	-	-	50
**Normal**	**-**	4	50	2	19	3	78
**Ataxia**	**Mild**	3	55	8	38	14	118
	**Moderate**	5	4	2	1	1	13
	**Severe**	-	7	-	-	-	7
**Steppage**	**Mild**	1	28	12	10	3	54
	**Moderate**	-	5	3	-	1	9
	**Severe**	-	11	1	3	8	23
**Longitudinal analysis**							
	**CANOMAD**	**CIDP**	**Hereditary neuropathy**	**IgM-MGUS**	**AN**	**N Total**	
**Control**	-	-	-	-	-	50	
**Change of ≥2 on MRCss**	-	83	7	23	8	121	
**Change of ≥4 on RODS**	-	56	2	29	6	93	

Classification according to their diagnosis, gait pattern and severity in the case of the analysis of differences in gait patterns. Classification according to their diagnosis and to the change in total score in MRCss or iRODS scale. Disease classification mainly included the inflammatory neuropathies CIDP, IgM-MGUS, CANOMAD, another AN and hereditary neuropathies. CANOMAD: chronic ataxic neuropathy, ophthalmoplegia, immunoglobulin M [IgM] paraprotein, cold agglutinins, and disialosyl antibodies; CIDP: chronic inflammatory demyelinating polyneuropathy; IgM-MGUS: monoclonal gammopathy of undetermined significance associated with IgM; AN: autoimmune nodopathy; MRCss: Medical Research Council sum score; iRODS: Inflammatory Rasch-built Overall Disability Scale.
